# Evaluation of Arterial Stiffness Parameters Measurement With Noninvasive Methods—A Systematic Review

**DOI:** 10.1155/crp/4944517

**Published:** 2024-12-19

**Authors:** Marta Maria Niwińska, Sławomir Chlabicz

**Affiliations:** Department of Family Medicine, Medical University of Białystok, Podlaskie Voivodeship, 15-054 Białystok, Poland

**Keywords:** cardiovascular risk, noninvasive devices, pulse wave velocity

## Abstract

**Objective:** Arterial stiffness, as determined by pulse wave velocity (PWV), is a recognized marker of cardiovascular risk. Noninvasive technologies have enabled easier and more accessible assessments of PWV. The current gold standard for measuring carotid–femoral PWV (cfPWV)—a reliable indicator of arterial stiffness—utilizes applanation tonometry devices, as recommended by the Artery Society Guidelines. The objective of this study was to compare the performance of various noninvasive arterial stiffness measurement methods, specifically the Mobil-O-Graph and SphygmoCor/SphygmoCor XCEL, and evaluate their alignment with the Artery Society Guidelines for accuracy and reliability.

**Methods:** A comprehensive search was conducted in the PubMed and Scopus databases to identify studies that compared and validated noninvasive PWV measurements, focusing on their repeatability. The search covered studies from inception through March 31, 2024. A total of 2092 papers were identified. Following the selection process, 21 studies met the inclusion criteria. Additionally, 2 more studies, not retrieved by the initial search but deemed relevant from other databases, were included. The included studies focused on populations with chronic diseases who were hemodynamically stable. Studies involving participants in specific conditions, such as pregnancy, hemodynamic shock, or undergoing stress tests, were excluded from the analysis.

**Results:** Several devices have been developed and validated for the noninvasive measurement of arterial stiffness, utilizing applanation tonometry (e.g., SphygmoCor, SphygmoCor XCEL) and cuff-based oscillometry (e.g., Arteriograph, Mobil-O-Graph). The analyses reviewed included studies using both invasive and noninvasive devices. A notable finding was the relative heterogeneity of study populations across different research, with variations in sample size, BMI, sex proportions, and age groups often falling short of guideline recommendations. In most of the included validation studies, the sample sizes were smaller than the minimum recommended by guidelines. Moreover, factors such as BMI, sex distribution, and age group sizes were inconsistent with established standards. Despite these limitations, validation studies comparing invasive and noninvasive methods consistently highlighted the superiority of cfPWV assessment devices. Applanation tonometry devices demonstrated smaller discrepancies in PWV measurements and better overall agreement with invasive methods than oscillometry-based devices. Three studies comparing the SphygmoCor XCEL with the standard SphygmoCor showed an excellent level of agreement, with one study confirming the SphygmoCor XCEL's superior adherence to validation criteria. Oscillometric devices showed a stronger reliance on algorithmic adjustments based on factors such as age and systolic blood pressure. This dependence likely contributes to the underestimation of PWV, particularly in populations with chronic diseases or other conditions promoting arterial stiffness. Despite this, oscillometric devices demonstrated lower PWV variability in short-term repeatability assessments.

**Conclusions:** More research on a larger population should be performed in order to introduce noninvasive devices into daily medical practice.

## 1. Introduction

Arterial stiffness is recognized as a reliable diagnostic marker for the early detection of cardiovascular disease (CVD), and its progression accelerates with age. Several factors, including chronic diseases, hypertension, unhealthy diet, and smoking, further contribute to the development of arterial stiffness [1].

Arterial stiffness is commonly assessed through the pulse wave velocity (PWV) parameter, a well-established and indirect measure of vascular rigidity. The pulse wave travels through the arterial system at a speed inversely related to the distensibility of the arterial walls: the higher the PWV, the lower the vessel's elasticity [2]. The relationship between arterial distensibility and PWV is described by the Bramwell–Hill equation, which indicates that arterial stiffness is defined by the relationship between arterial pressure and the cross-sectional area of the vessel [3].

(1) $$\begin{array}{c} \text{PWVest}=\sqrt{\frac{A}{\rho }\frac{\Delta P}{\Delta A}}=\sqrt{\frac{\Delta P}{\rho }\frac{A}{\Delta A},} \end{array}$$

• *ρ* represents the density of the blood;
• A is the mean area of the blood vessel;
• Δ*A* is the difference between the maximum and minimum area of the blood vessel during a cardiac cycle;
• Δ*P* is the difference between the central systolic and diastolic pressures;
• PWVest is the estimated PWV obtained from the measurement of pulsatility (Δ*A*/*A*) and pressure variation (Δ*P*).

However, in clinical practice, PWV is mostly defined by the distance between 2 measurement sites and the transition time of the pulse wave from the proximal to the distal measurement site. This is explained by the following formula [4]:

(2) $$\begin{array}{c} \text{PWV}=\frac{\Delta x}{\Delta t}, \end{array}$$

• Δ*x* is the distance between 2 measurement sites;
• Δ*t* is the transit time.

Arterial stiffness not only increases cardiovascular risk but is also associated with various adverse cardiovascular outcomes. Additionally, it plays a significant role in the development of dementia, stroke, and chronic kidney disease [5–10]. The predictive value of arterial stiffness is particularly strong in individuals with hypertension and those at higher baseline cardiovascular risk.

Incorporating PWV into risk models improves their predictive accuracy and helps reclassify individuals' risk for future CVD events, compared to models that only include traditional risk factors. Aortic PWV, specifically, can enhance the identification of high-risk populations who may benefit from more intensive treatments and improved management of cardiovascular risk factors [6].

Despite the high predictive value of PWV, there are currently no clear guidelines on which specific patient groups should undergo PWV measurement [11, 12]. Various devices are available for determining PWV, with invasive pressure catheter recordings being the most accurate. However, this method is limited to patients undergoing cardiac catheterization.

Noninvasive yet highly precise methods, such as magnetic resonance imaging (MRI), are also available, but their high costs and logistical complexity make them impractical for widespread use in the general population. More accessible noninvasive methods that can be used in an office setting include oscillometric devices like the Mobil-O-Graph and applanation tonometry devices like SphygmoCor [13].

### 1.1. Devices

The Mobil-O-Graph uses a cuff-based method to estimate PWV by recording the pressure wave at a single point on the brachial artery. PWV is calculated from the time difference between the forward and reflected waves, which are derived using the ARCSolver algorithm. This algorithm integrates age, central systolic blood pressure (BP), and data from peripheral pulse wave analysis into a mathematical model to estimate PWV [14].

During SphygmoCor XCEL cfPWV measurement, the carotid pulse is recorded with an applanation tonometer and the femoral pulse with a cuff placed on the upper thigh, simultaneously. The transition time of the pulse wave from the carotid to the femoral artery is measured. The transition distance of the pulse wave is measured from the tonometry point of the carotid artery to the suprasternal notch then, from that point to the top of the cuff on the thigh [14]. Using a cuff seems to be more effortless for the user, due to the possible limitations associated with tonometry such as difficulties in obtaining a high-quality femoral pulse in some groups of patients and operator dependency of the peripheral signal acquisition [4, 15].

However, the effective femoral pressure measurement location is more distal than with a tonometer, requiring a correction equation to convert the resulting PWV value to a value that corresponds to cfPWV [4].

The SphygmoCorCvMS and SphygmoCor XCEL devices both measure aortic pressure waveform parameters and carotid–femoral PWV (cfPWV), but they use different methods for acquiring peripheral arterial waveforms.

For the SphygmoCorCvMS, the carotid and femoral pulses are sequentially captured using applanation tonometry. During the measurement, the transit time from the R-wave of a simultaneously recorded electrocardiogram (ECG) to the foot of the carotid and femoral pulses is measured. The difference between these two transit times is then divided by the distance measured from the body surface, estimating the arterial path length to calculate cfPWV [15]. The transit time is determined using the intersecting tangent method (ITM) [16].

In the SphygmoCor XCEL, cfPWV is measured by recording the carotid pulse with an applanation tonometer and the femoral pulse with a cuff placed on the upper thigh, simultaneously. The transit time of the pulse wave between the carotid and femoral arteries is then measured. The pulse wave travel distance is calculated from the carotid tonometry point to the suprasternal notch and from there to the top of the thigh cuff [14].

Using a cuff for femoral pulse acquisition tends to be easier for the operator due to the challenges of using tonometry in certain patient populations, such as difficulty obtaining a high-quality femoral pulse and the operator dependency of the signal acquisition [4, 15]. However, since the cuff measures the pulse at a more distal location than tonometry, a correction equation is applied to adjust the PWV value to match the standard cfPWV measurement [4].

According to the 2023 ESC/ESH Guidelines for the management of arterial hypertension, cfPWV is the gold standard for assessing large artery stiffness. A PWV value exceeding 10 m/s indicates organ damage, significant impairment of aortic function, and an increased cardiovascular risk, particularly in middle-aged hypertensive patients [11].

The American Heart Association (AHA) guidelines also highlight the need for further research on PWV assessment in high-risk populations, along with additional validation studies. This recommendation is supported by the AHA guidelines for cardiovascular risk assessment in asymptomatic adults and the 2015 AHA recommendations for improving and standardizing vascular studies of arterial stiffness [17, 18].

Validation of PWV measurement methods should follow the ARTERY Society's validation guidelines. The ARTERY Society guidelines, along with the latest “Recommendations for Validation of Noninvasive Arterial Pulse Wave Velocity Measurement,” state that simultaneous applanation tonometry of the carotid and femoral arteries is the noninvasive reference method for cfPWV measurement in studies [4, 19].

## 2. Aim of the Study

The aim of this systematic review is to evaluate studies that utilize noninvasive PWV devices, focusing on three key aspects: comparison of PWV results, measurement repeatability, and factors influencing PWV assessment. Additionally, the review seeks to determine the level of agreement in validation studies in accordance with the ARTERY Society guidelines and the recommendations published by AHA [17, 19].

## 3. Methods

**Figure 1:**
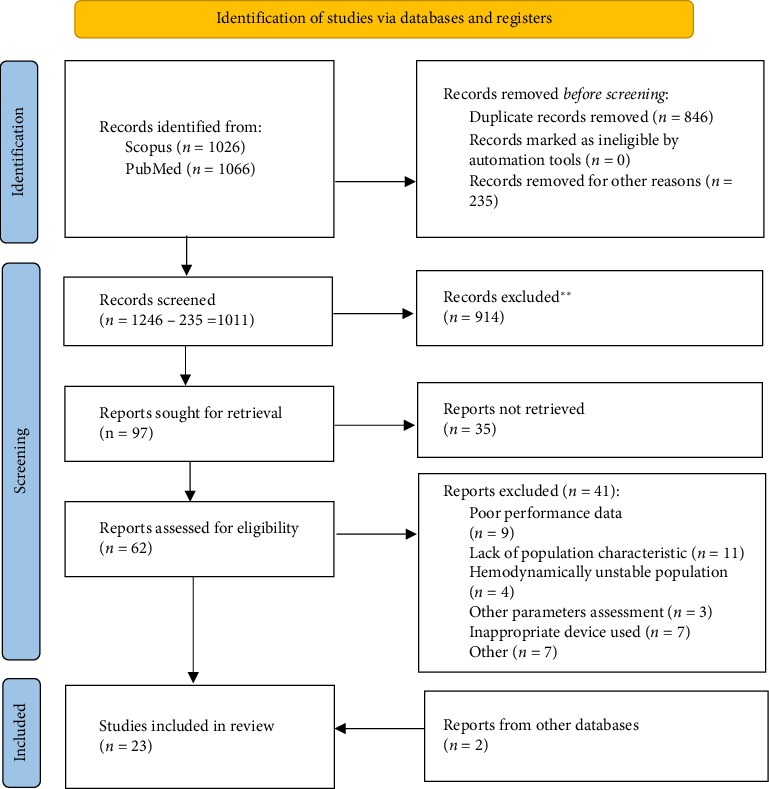
PRISMA flowchart depicting the search strategy employed for this review.

This systematic review was conducted in accordance with the Preferred Reporting Items for Systematic Reviews and Meta-Analyses (PRISMA) guidelines (Figure 1). Comparative studies of noninvasive devices were analyzed, with validation of these devices carried out following the ARTERY Society guidelines for assessing arterial stiffness and AHA recommendations for improving and standardizing vascular research on arterial stiffness [[17, 19], Table 8]. The studies included in this review were published prior to the release of the updated 2024 Recommendations for Validation of Noninvasive Arterial Pulse Wave Velocity Measurement Devices [[4], Table 9]. Consequently, the validation was performed in accordance with the earlier criteria [19].

## 4. Search Strategy

The PubMed and Scopus databases were searched to gather relevant studies for further selection. The search utilized the terms and phrases outlined in Table 1. A total of 2092 records were identified. EndNote 21 software was employed to detect duplicates and unsuitable articles. Before screening the titles and abstracts, 846 duplicates were removed. An additional 246 records were excluded for various reasons, leaving 1011 articles for screening.

Upon analyzing the abstracts and titles, 914 records were eliminated for being irrelevant to the research question and outcomes, resulting in 97 studies assessed for further retrieval. After a thorough search, 35 full texts were not available. The remaining 62 records underwent eligibility assessment. After full-text analysis, 21 articles from PubMed and Scopus were included in this review. Additionally, 2 more studies—identified through a review of the bibliographies of relevant papers and not retrieved by the original search criteria—were included. A comprehensive search was conducted from study inception to March 30, 2024.

### 4.1. Inclusion and Exclusion Criteria

The inclusion criteria for this systematic review primarily focused on studies that considered PWV as a parameter for arterial stiffness in validation studies or comparative analyses. Studies that primarily addressed other arterial stiffness parameters, such as central BP or augmentation index, were excluded. Acceptable noninvasive devices included the SphygmoCor, SphygmoCor XCEL, and Mobil-O-Graph, as well as devices with similar mechanisms and algorithms, such as the Tel-O-Graph and Arteriograph (oscillometric devices).

Reference methods for validation included the SphygmoCor and invasive PWV (PWVinv) assessments. The types of articles included were comparative analyses of specific methods (either noninvasive to noninvasive or noninvasive to invasive) and validation studies that adhered to ARTERY Society guidelines. Additionally, research focusing on the repeatability of measurements and factors affecting PWV results was included. An accurate description of the procedures performed was also a requirement for inclusion.

There were no specific requirements regarding the age, origin, or publication date of the studies included in the analysis. Studies involving hemodynamically stable subjects with conditions such as chronic kidney disease, hypertension, and diabetes were accepted. In contrast, studies involving patient groups with specific systemic states—including pregnancy, severe heart failure, sepsis, or other conditions that significantly disrupt normal hemodynamics—were excluded. Analyses focused solely on PWV assessment during stress tests were also omitted.

Additional restrictions included the exclusion of studies that performed PWV assessments with devices with mechanism and algorithm other than SphygmoCor, SphygmoCor XCEL and Mobil-O-Graph, lacked relevant performance data, or were conducted on animal subjects. Studies that did not report any numerical PWV values for the methods used were also excluded. The study type (e.g., randomized vs. cross-sectional) did not affect inclusion. Papers written in a language other than English were excluded. In some cases, studies that did not provide a comparison to another device or failed to clearly explain the established diagnostic modality in the abstract were also excluded after full-text analysis.

### 4.2. Study Characteristics, Data Extraction, and Risk of Bias

Characteristics of the included studies (state of health, country, and origin) are presented in Table 2.

For all study types, the following data were extracted: population size (including the percentage of women), a description of the population (mean age and body mass index [BMI]), and the device used for the entire population, as well as for any subgroups. For the included studies, mean PWV and mean differences were reported. In studies assessing repeatability, PWV results from individual measurements were also extracted. The main findings, extracted data, and conclusions of each study and other reports required for validation are summarized and illustrated in Tables 3, 4, 5, 6, 7.

To evaluate the quality of the included studies regarding design, content, clarity of results, and usefulness for interpreting the systematic review, the Newcastle–Ottawa Scale (NOS) modified for cross-sectional studies was utilized. Each study was rated from 0 to 10 points, with only two studies achieving 8 points. Detailed information on the criteria for study assessment is provided in the supporting information

To minimize potential bias, the review predominantly included studies utilizing the SphygmoCor/SphygmoCor XCEL and Mobil-O-Graph devices. The reference methods for validation included applanation tonometry and PWVinv assessment.

Given the heterogeneity of the included studies (e.g., variations in age, sex, BMI, and health status), the results were synthesized through a narrative description, focusing on disease burden and the devices used. The present review is organized into several sections: validation of noninvasive versus invasive methods, comparison and validation of SphygmoCor XCEL and SphygmoCor results, comparisons or validation studies of noninvasive PWV (including SphygmoCor XCEL), noninvasive PWV measurement in populations with chronic diseases, and measurement repeatability.

## 5. Results

The results of selected noninvasive PWV studies are presented below. Comparative analyses and studies evaluating repeatability and the impact of various factors on PWV have met the established criteria for inclusion in this review.

The ARTERY Society guidelines [4, 19] outline the criteria for validating noninvasive PWV assessment devices. The gold standard methods for validation include simultaneous carotid–femoral artery applanation tonometry and the assessment of simultaneous pressure waveforms recorded invasively using high-fidelity pressure sensors positioned just above the aortic valve and just above the aortic bifurcation. Detailed information regarding subject selection criteria for validation studies and measurement requirements for reference standards can be found in Table 8 [19]. The updated criteria for subject selection are illustrated in Table 9 [4].

Not all of the included papers were validation studies. Only one study [20] demonstrated nearly complete adherence to the guidelines [19] (Table 4). The main discrepancies noted involved the BMI of the included patients, with only one validation study considering a BMI greater than 30 kg/m^2^ as an exclusion criterion [20]. A minimum sample size of 83 individuals in validation studies was achieved in eight studies [2, 20–26]. Furthermore, the age range divisions suggested by the guidelines [19] were reported, and the minimum number of individuals recommended in each age range was met in two papers [20, 22] (Tables 3 and 4). Only six studies reached the necessary minimum of 40% representation of each sex within the studied population [20, 23–27].

### 5.1. Noninvasive Against Invasive Method Validation

Noninvasive PWV was validated against PWVinv in five studies (Table 3). In a comprehensive analysis involving various noninvasive devices, those assessing cfPWV demonstrated better agreement with PWVinv, showing a mean difference of −0.61 ± 2.57 m/s for SphygmoCor. In contrast, oscillometric devices, such as Mobil-O-Graph, exhibited weaker agreement with PWVinv, particularly at higher PWV values, with a mean difference of −1.01 ± 2.54 m/s. Additionally, a weak relationship between SphygmoCor and Mobil-O-Graph PWV was observed, with a mean difference of −0.40 ± 2.24 m/s [2].

In the study by Weber et al., SphygmoCor was validated against PWVinv, and different methods for noninvasive estimation of pulse wave travel distance were discussed. This study reported the best agreement when the path length was estimated using the notch-femoral carotid-notch method [21]. In a subsequent study, these findings were confirmed, revealing a highly significant correlation (*p* < 0.0001) between PWVinv and cfPWV when using the notch-femoral carotid-notch path length estimation. However, the Bland–Altman analysis indicated a mean PWV difference of 0.8 ± 2.1 m/s, suggesting poor accuracy [22].

A study conducted on a pediatric and young adult population found a positive linear relationship between both invasive measurements (ascending aorta and entire central aorta PWV) and Mobil-O-Graph measurements across the overall group. Notably, better agreement was observed with the ascending aorta PWV, with a mean difference of 0.44 ± 0.55 m/s [28]. An acceptable agreement between Mobil-O-Graph PWV and PWVinv was reported in a study by Hametner et al., which included 120 patients; the mean difference between brachial cuff-derived and intra-aortic PWV values was 0.43 ± 1.24 m/s [23].

### 5.2. SphygmoCor XCEL and SphygmoCor Results Comparison and Validation

The validation of SphygmoCor XCEL was evaluated in three studies (see Table 4). An analysis conducted on a pediatric and adolescent population demonstrated a strong correlation (*p* < 0.001) and acceptable agreement between the cfPWV measurements obtained from SphygmoCor XCEL and those from SphygmoCor [27]. In the adult population, the conformity of results from SphygmoCor XCEL and SphygmoCor was also confirmed. Notably, the analysis by Butlin et al. revealed an excellent level of accuracy [20, 29], with all ARTERY Society criteria being met.

### 5.3. Noninvasive PWV Comparison or Validation Study (Including SphygmoCor XCEL)

Noninvasive PWV comparison and validation were discussed in 12 studies (Table 5). An analysis conducted on a South African population revealed that oscillometric PWV measurements were significantly lower than those obtained with SphygmoCor XCEL, with this discrepancy being particularly pronounced among the young adult population. Interestingly, this observation was not found among children [30].

In a comparative study examining 24-h mean values of arterial stiffness parameters obtained using a 24-h ambulatory BP monitoring device (Mobil-O-Graph) versus applanation tonometry, the 24-h mean values of PWV derived from Mobil-O-Graph were significantly lower than the office SphygmoCor values (*p* < 0.001) [31].

The study by del Giorno et al. compared oscillometric and tonometric devices, revealing that oscillometric PWV tended to underestimate arterial stiffness in younger subjects while overestimating it with increasing age. Discrepancies in results were noted in patients with conditions known to increase arterial stiffness. Specifically, analysis of patients with confirmed vascular damage (PWV ≥ 10 m/s) showed a greater difference in mean values between oscillometric PWV and cfPWV compared to the subgroup of subjects with a PWV < 10 m/s [25].

In another study evaluating SphygmoCor and Mobil-O-Graph PWV measurements, oscillometric results were significantly higher than those obtained through tonometry in cases of low-grade atherosclerosis and lower mean PWV. However, as the severity of arterial stiffness increased, this trend reversed; oscillometric results became significantly lower, leading to an underestimation of arterial stiffness [32].

### 5.4. Noninvasive PWV Measurement in Population With Chronic Diseases

Among the studies focusing on populations with chronic comorbidities, four studies were included (Table 6). Three of these studies examined patients undergoing peritoneal dialysis. The survey conducted by Sarafidis et al. compared aortic parameters and PWV measurements obtained from Mobil-O-Graph and SphygmoCor, revealing that oscillometric measurements underestimated PWV (*p* < 0.01) [33]. The preliminary study by Vaios et al. found good agreement between the arterial stiffness indices estimated by Mobil-O-Graph and SphygmoCor in a cohort of 27 peritoneal dialysis patients [34].

Additionally, an analysis focusing on the determinants of oscillometric PWV in 81 patients with end-stage kidney disease concluded that the strong age dependence of oscillometric PWV limits its validity as a marker for detecting premature vascular aging in this population [35]. In the case of hypertensive patients, the PWV obtained through oscillometry was significantly lower than the tonometric PWV measured with SphygmoCor [36].

### 5.5. Factors Affecting PWV

Two studies focused on identifying factors that influence PWV and PWVinv (see Table 6). The analysis conducted by Schwartz et al. indicated that the PWV measured by Mobil-O-Graph is nearly completely explained by age and systolic BP [37]. In another study involving population with type 2 diabetes mellitus, it was found that age, systolic office BP, duration of diabetes, serum LDL-cholesterol levels, and waist circumference were all significantly related to the PWV measured by SphygmoCor [38].

### 5.6. Measurement Repeatability

Two studies assessed the repeatability of PWV measurements, while two others evaluated factors influencing this repeatability (see Table 7). A comparative analysis of Tel-O-Graph, which utilizes the same mathematical algorithm as Mobil-O-Graph, against SphygmoCor demonstrated a significantly lower coefficient of variation (CV) for the oscillometric device (2.38 ± 6.13% for Tel-O-Graph vs. 6.30 ± 4.33% for SphygmoCor; *p* < 0.05) [26]. Grillo et al. investigated the short-term repeatability of noninvasive devices measuring cfPWV and PWV and concluded that overall repeatability was good (CV < 10% for all devices); however, it varied across different devices and PWV values. Oscillometric methods exhibited a lower percentage of variability (CV = 3.5% for Mobil-O-Graph; CV = 9.5% for SphygmoCor). Notably, when PWV values exceeded 10 m/s, significantly higher variability in SphygmoCorcfPWV was observed (CV = 3.52% for Mobil-O-Graph; CV = 10.29% for SphygmoCor). The differences in PWV between repeated measurements were influenced by short-term variations in mean BP and heart rate for Mobil-O-Graph [14].

Silva et al. conducted research to evaluate the correlation between short-term BP variance parameters and Mobil-O-Graph PWV in individuals suspected of hypertension who were undergoing treatment. Their study revealed a strong correlation between PWV and systolic BP variance [39]. Similarly, Podrug et al. found that mean arterial pressure influenced PWV measurements obtained through both oscillometric and tonometric methods. However, the oscillometric results were more significantly affected by factors such as age, height, sex, and current heart rate [40].

## 6. Discussion

In this systematic review, the accuracy of PWV measurements was established in 10 studies in accordance with the 2010 Artery Society Guidelines and the 2015 AHA Scientific Statement [17, 19]. One study was published prior to the establishment of these guidelines; however, it demonstrated general conformity to the inclusion and exclusion criteria, as well as the hemodynamic stability and age distribution of the enrolled individuals [21].

The studies included in this review were published before the updated guidelines, meaning none of them were validated according to the 2024 Recommendations for Validation of Noninvasive Arterial Pulse Wave Velocity Measurement Devices [4]. Discrepancies among the guidelines pertained to sample size, age group distribution, and the exclusion of participants based on BMI, all of which could potentially lead to biased results [[4, 19], Tables 8 and 9].

PWV assessment during cardiac catheterization is considered the gold standard and the most reliable method for PWV validation [4, 19]. However, this procedure is limited to patients referred for coronary angiography, which may restrict sample size. Given its invasiveness and limited availability, noninvasive measurements appear promising but come with certain limitations that may impair PWV measurement accuracy [2, 4, 19].

Applanation tonometry is regarded as the most accurate noninvasive method for assessing cfPWV. The cfPWV obtained using this technique is measured directly and is therefore considered to have the greatest predictive value [19]. Nevertheless, tonometric devices and cfPWV assessments face some limitations. For instance, results depend on the external carotid–femoral distance, which does not accurately reflect true aortic length [4, 12, 14, 17]. Higher BMI and abdominal obesity can hinder accurate estimation of the distance traveled by the pulse wave due to increased body surface area. Consequently, the Artery Society does not recommend measurements in such populations. Current guidelines disqualify participants with a BMI greater than 40 kg/m^2^ [4]. In contrast, the 2010 recommendations excluded participants with a BMI over 30 kg/m^2^ [19]. In the majority of included validation studies, participants with a BMI over 30 kg/m^2^ were not excluded [2, 21–27, 29, 33], which likely affected the reliability of the results.

A further limitation of applanation tonometry is that it includes portions of both the carotid and femoral arteries in its measurements. These arteries exhibit a reduction in elastic components and an increase in muscular components. While PWV in the aorta demonstrates a significant exponential increase with age, PWV in muscular arteries rises weakly and linearly [2].

When considering the SphygmoCor XCEL, the transit time measured between the carotid site and the cuff-acquired femoral artery signal point requires even more correction compared to the original SphygmoCor. The pulse detection location obtained by the cuff encompasses a larger segment of the femoral artery, necessitating adjustments to mathematically eliminate this additional section from the PWV acquisition [20].

A significant drawback of each cfPWV assessment device is that they do not account for pulse wave travel in the aortic arch and ascending aorta. These sections of the aorta are critical to its buffering function and undergo substantial changes with aging [12].

cfPWV measurements can also be influenced by errors related to height and body composition differences. In a study involving both adults and children in South Africa, SphygmoCor XCEL and Mobil-O-Graph PWV were compared, revealing an overestimation of PWV by SphygmoCor XCEL in the pediatric group. This discrepancy is attributed not only to the height of the subjects but also to variations in body composition, such as differences in trunk and leg length across different age groups. This issue is further compounded by the prevalence of malnutrition and stunting among African children [28].

Despite these limitations, most of the comparative and validation studies presented indicate a compatibility between cfPWV and PWVinv. Moreover, all of the included validation studies for SphygmoCor XCEL demonstrated satisfactory results, which may facilitate the broader use of femoral-cuff-based devices due to their ease of use.

Oscillometric devices derive PWV based on the analysis of peripheral pressure waves obtained from a single site, combined with mathematical algorithms that incorporate factors such as age, sex, height, and current BP. Consequently, the PWV produced by this method is estimated rather than measured, as it does not account for the carotid–femoral distance and pulse wave travel time [14].

Therefore, a significant issue with these devices is their strong dependence on age and systolic BP. While these two factors correlate with overall cardiovascular risk, they do not provide additional prognostic information, particularly in populations with chronic diseases and premature vascular stiffening. This relationship was highlighted in research conducted on patients with end-stage renal disease, which focused on the association of 24-h Mobil-O-Graph PWV with coexisting factors. The strongest correlations were observed with age and current systolic BP [35]. Other studies on populations with chronic diseases also demonstrated a strong dependence of PWV on algorithmic data, beyond other significant cardiovascular risk factors [37].

In a large study by Salvi et al., which compared different noninvasive PWV methods to PWVinv, a greater reliability was revealed for devices that measure cfPWV [2]. PWV values obtained through cuff-based devices may predominantly reflect changes in BP levels rather than actual alterations in arterial distensibility. Consequently, algorithm-based systems, such as Mobil-O-Graph, may not be adequate for clinical trials, epidemiological studies, or research involving subjects at high cardiovascular risk—conditions in which factors beyond age and BP fluctuations are crucial.

In summary, these devices may serve as useful diagnostic tools in the general, comorbidity-free population to assess cardiovascular risk based on classic risk factors. This was evidenced in a study conducted on pediatric and young adult populations, which demonstrated good conformity of results from Mobil-O-Graph to PWVinv. This conformity may be attributed to the selection of a younger population that is less burdened by chronic diseases.

An undeniable advantage of oscillometric devices is their repeatability. A comparative analysis of Tel-O-Graph with SphygmoCor showed a significantly lower CV for the former. Furthermore, this study indicated no significant differences in single PWV measurements or when averaging three values measured with Tel-O-Graph. These results suggest that due to the low variation between measurements, a single PWV assessment using Tel-O-Graph is sufficient for calculating a reliable and clinically relevant cfPWV value [26].

In a comparative study assessing short-term repeatability of noninvasive devices measuring PWV, Grillo et al. found that both cfPWV-determining devices and oscillometric methods generally exhibited low coefficients of variation. However, cuff-based methods demonstrated a lower percentage of variability. Devices assessing cfPWV had lower repeatability, especially for PWV results exceeding 10 m/s, with discrepancies often observed in higher PWV values. Tonometry determines the transit time of the pulse wave over a specific distance; from a physical standpoint, a shorter transit time correlates with a higher PWV. Notably, variability in heart rate and mean arterial pressure did not influence result variability. In contrast, for oscillometric devices, mean arterial pressure and heart rate did not significantly affect the obtained PWV values, except for Mobil-O-Graph [14].

## 7. Conclusions

Arterial stiffness is widely recognized as an independent predictive factor for cardiovascular events in both higher risk and general populations. Devices that measure cfPWV are generally considered more reliable for assessing arterial stiffness and, consequently, cardiovascular risk. However, it is crucial to consider the repeatability of these devices, as they tend to exhibit higher variability compared to oscillometric techniques.

Key limitations affecting reliability include small and heterogeneous sample sizes, as well as limited conformity with existing guidelines in validation studies. To address the discrepancies identified in this review, further research should be conducted on larger populations that adhere to current recommendations.

## Figures and Tables

**Table 1:** Full list of search terms used in the review.

No	Search term
1	Tonometric and oscillometric pulse wave velocity measurement methods
2	Mobil-O-Graph PWV assessment
3	SphygmoCor PWV measurement
4	Mobil-O-Graph vs. SphygmoCor
5	Non-invasive PWV assessment/measurement
6	Tonometry vs. oscillometry
7	Comparison of SphygmoCor and Mobil-O-Graph
8	Non-invasive carotid–femoral pulse wave velocity
9	Oscillometric pulse wave velocity

**Table 2:** Characteristics of the study population of included papers.

Author	Subjects	Age	BMI	Population	Origin	Country
Salvi et al.	102 (30% women)	65 ± 13	—	Not admitted (NA)	NA	Italy
Weber et al.	135 (34.1% women)	60.3 ± 12.5	—	Suspection for CAD⁣^∗∗^	NA	Austria
Weber et al.	915 (17.8% women)	61.0 ± 8	28.3	Suspection for CAD⁣^∗∗^	NA	Austria
Walser et al.	60 (58.3% women)	16.6 ± 8.2	19.6 ± 4.8	Atrial septal defect⁣^∗^	NA	Germany
Hametner et al.	120 (9.2% women)	61.8 ± 10.8	—	NA	NA	England
Stabouli et al.	68 (52.9% women)	11.5 ± 3.7	19.1 ± 53.7	NA	NA	Australia, Italy, France, England
Hwang et al.	26 (38.5% women)	44 ± 4	25.2 ± 0.7	NA	Asians, Caucasians and Spain	NA
Bultin et al.	88 (49% women)	46 ± 20	23 ± 3	NA	NA	Australia, Italy, France, England
Kolkenbeck-Ruh et al.	112 (100% women)	Adults: 29 Children: 7	Adults: 30.2 Children:16	NA	South Africa	Republic of South Africa
Berukstis et al.	82 (46.3% women)	56.6 ± 7.5	32.9 ± 4.9	NA	NA	Austria
Van Dijk et al.	344 (42% women)	72.5 ± 5.5	26.5 ± 3.4	HT⁣^∗^, diabetes mellitus type 2 (DM2)⁣^∗^, hypercholesterolemia⁣^∗^, cardiac diseases⁣^∗^	NA	Netherlands
Del Giorno et al.	1162 (56.0% women)	52	—	DM2⁣^∗^, hypertension (HT)⁣^∗^	NA	Switzerland
Bia et al.	3619	33.9 ± 24.2	24.1 ± 6.0	DM2⁣^∗^, HT⁣^∗^	NA	Uruguay
Safardis et al.	73 (39.7% women)	61.7 ± 15.3	25.6 ± 4.9	End-stage kidney disease (ESKD)⁣^∗∗^, DM2⁣^∗^, HT⁣^∗^	NA	Greece
Vaios et al.	27	62.5 ± 15.6	—	ESKD⁣^∗∗^, DM2⁣^∗^, HT⁣^∗^	NA	Greece
Vaios et al.	81 (34.8% women)	61.3 ± 16.3	26.2 ± 4.3	ESKD⁣^∗∗^, HT⁣^∗^, NA⁣^∗^	NA	Greece
Barosso et al.	27 (64% women)	50.8 ± 15	27.3 ± 4.8	HT⁣^∗∗^	NA	Brazil
Schwartz et al.	234 (66.2% women)	52.8 ± 9.9	27.9 ± 5.5	HT⁣^∗∗^	Black/African- American 8.5%, Caucasian	
Staef et al.	266 (34.6% women)	61.0 ± 8.1	30.5 ± 4.2	DM2⁣^∗∗^	NA	Germany
Reshetnik et al.	89 (40.4% women)	48.8 ± 19.1	24.7 ± 4.7	NA	NA	Germany
Grillo et al.	102 (29.4% women)	65 ± 13	—	NA	NA	Italy
Silva et al.	649 (49.4% women)	47.7 ± 14.2	28.8 ± 5.8	NA	NA	NA
Podrug et al.	35	41	27.3	HT⁣^∗^	NA	Croatia

**Table 3:** Studies analyzed with reference to the ARTERY Society guidelines.

Device (referenced italics)	Subjects	Mean age	BMI	Transit time	PWV device	PWV referenced device	PWV mean difference	Accuracy	General conclusion	Reference
*Invasive* *SphygmoCor* Mobil-O-Graph	102 (30% women)	65 ± 13	—	ITM	—	—	0.6 ± 2.6 (SphygmoCor 1.0 ± 2.5 (Mobil-O-Graph))	Poor Poor	cfPWV assessing devices are more reliable	Salvi et al.
SphygmoCor *Invasive*	135 (34.1% women)	60.3 ± 12.5(age range reported and respected)	—	ITM	8.7 ± 2.2	8.5 ± 2.4	−0.2 ± 1.55	Poor	Better agreement with invasive method, when subtracking path length	Weber et al.
SphygmoCor *Invasive*	915 (17.8% women)	61.0 ± 8	28.3	ITM	8.1 (7.0–9.6)	8.3 (7.1–9.8)	0.1 ± 2	Poor	Better agreement with invasive method, when subtracking path length. Acceptable agreement of Mobil-O-Graph with invasive method	Weber et al.
*Invasive* Mobil-O-Graph	60 35 (58.3% women)	16.6 ± 8.2	19.6 ± 4.8	Not assessable	4.44 (0.56)	Ascending aorta PWVinv: 4.88 ± 0.68 Entire central aorta PWVinv: 5.18 ± 0.98	Ascending aorta PWVinv: 0.44 ± 0.55 entire central aorta PWVinv: 0.74 ± 0.84	Excellent Acceptable	Excellent agreement with invasive method	Walser et al.
Invasive Mobil-O-Graph (ARCSolver algorithm)	20 (9.2% women)	61.8 ± 10.8 (age range reported)	—	Not assessable	8.5 ± 2.11	8.9 ± 1.7	0.4 ± 1.2	Acceptable	Good agreement with invasive method	Hametner et al.

**Table 4:** Studies analyzed with reference to the ARTERY Society guidelines.

Device (referenced italics)	Subjects	Mean age	BMI	Transit time	PWV device	PWV referenced device	PWV mean difference	Accuracy	General conclusion	Reference
*SphygmoCor* SphygmoCor XCEL	68 (52.9% women)	11.5 ± 3.7	19.1 ± 5 3.7	ITM	4.75 ± 0.8	4.85 ± 0.8	0.1 ± 0.5	Excellent	SphygmoCor XCEL provides equivalent cfPWV results as SphygmoCor	Stabouli et al.
*SphygmoCor* SphygmoCor XCEL	26 (38.5% women)	44 ± 4	25.2 ± 0.7	ITM	6.3 ± 0.3	6.5 ± 0.3	0.2 ± 0.2	Excellent	SphygmoCor provides equivalent PWV results as SphygmoCor.	Hwang et al.
*SphygmoCor* SphygmoCor XCEL	88 (49% women)	46 ± 20 (age range reported and respected)	23 ± 3 (BMI > 30 excluded)	ITM	7.04 ± 1.7	7.02 ± 1.9	0.01 ± 0.71	Excellent	SphygmoCor XCEL provides equivalent cfPWV results as SphygmoCor	Bultim et al.

**Table 5:** Studies analyzed with reference to the ARTERY Society guidelines.

Device (referenced italics)	Subjects	Mean age	BMI	Transit time	PWV device	PWV referenced device	PWV mean difference	Accuracy	General conclusion	Reference
SphygmoCor Xcel Mobil-O-Graph	112 (100% women)	Adults: 29 Children: 7	Adults: 30.2 Children: 16		Adults: 5.9 (5.0–8.1) Children: 4.3 (4.1–4.6)	Adults: 7.3 (6.4–8.5) Children: 4.3 (3.9–4.6)	—	Not assessable	Underestimation by Mobil-O-Graph	Kolkenbeck-Ruh et al.
*SphygmoCor,* Mobil-O-Graph	82 (46.3% women)	56.6 ± 7.5	32.9 ± 4.9	ITM	8.7 ± 1.3	10.6 ± 2.6	1.8 ± 2.1	Not assessable	Underestimation by Mobil-O-Graph	Berukstis et al.
*SphygmoCor* Arteriograph	344 (42% women)	72.5 ± 5.5	26.5 ± 3.4	ITM	> 12 ms: 10.3 ± 2.0 < 12 m/s: 9.9 ± 2.3	> 12 m/s: 16.1 ± 4.1 < 12 m/s: 10.1 ± 2.0	> 12 m/s - 6.1 ± 4.5 < 12 m/s- 0.29 ± 2.8	Poor Poor	Results differ in different values	Van Dijk et al.
*SphygmoCor* Mobil-O-Graph	1162 (56.0% women)	52	—	ITM	Overall: 7.6 ± 1.9 > 10 m/s: 10.0 ± 2.1 < 10 m/s: 7.4 ± 1.7	Overall: 7.3 ± 1.8 > 10 m/s: 11.4 ± 1.4 < 10 m/s: 6.9 ± 1.3	Overall: −0.3 ± 0.05 > 10 m/s: 1.4 ± 0.2 < 10 m/s: 0.5 ± 0.05	Excellent Poor Excellent	Accuracy depends on mean PWV and age	Del Giorno et al.
SphygmoCor Mobil-O-Graph	3619 (% of each gender not involved)	33.9 ± 24.2	24.1 ± 6.0	ITM	5.7 ± 2.1	7.4 ± 2.5	—	Not assessable	Oscillometry depends mainly on age	Bia et al.
*SphygmoCor* Mobil-O-Graph	73(39.7% women)	61.7 ± 15.3	25.6 ± 4.9	ITM	9.5 ± 2.1	10.3 ± 3.4	0.8 ± 2.3	Poor	Underestimation by Mobil-O-Graph	Sarafidis et al.

**Table 6:** Studies analyzed with reference to the ARTERY Society guidelines.

Device (referenced italics)	Subjects	Mean age	BMI	Transit time	PWV device	PWV referenced device	PWV mean difference	Accuracy	General conclusion	Reference
*SphygmoCor* Mobil-O-Graph	27	62.5 ± 15.6	—	ITM	9.5 ± 2.1	10.1 ± 3.1	—	—	Acceptable agreement in PWV assessment by both devices in peritoneal dialysis patients	Vaios et al.
Mobil-O-Graph	81 (34.8% women)	61.3 ± 16.3	26.2 ± 4.3	—	Overall: 9.0 ± 2.2 > 10 m/s: 11.1 ± 0.8 < 10 m/s: 7.5 ± 1.5	—	—	Not assessable	Oscillometry depends mainly on age, blood pressure and heart rate variation, thus limits validity of arterial stiffness assessment	Vaios et al.
*SphygmoCor* Mobil-O-Graph	27 (64.0% women)	50.8 ± 15	27.3 ± 4.8	ITM	7.4 ± 1.4	8.4 ± 1.6	—	Not assessable	Underestimation by Mobil-O-Graph	Barosso et al.
*SphygmoCor* Mobil-O-Graph	79 (66.2% women)	52.8 ± 9.9	27.9 ± 5.5	ITM	7.6 ± 1.3	7.7 ± 1.7	Not included	Not assessable	PWV is explained by age and SBP	Schwartz et al.
*SphygmoCor*	266 (34.6% women)	61.0 ± 8.1	30.5 ± 4.2	ITM	—	8.9 ± 1.8	—	Not assessable	cfPWV is associated with age, SBP, diabetes duration, LDL, waist circumference	Staef et al.

**Table 7:** Studies analyzed with reference to the ARTERY Society guidelines.

Device (referenced italics)	Subjects	Mean age	BMI	Transit time	PWV device	PWV referenced device	PWV mean difference	Accuracy	General conclusion	Reference
*SphygmoCor* Tel-O-Graph	89 (40.4% women)	48.8 ± 19.1	24.7 ± 4.7	ITM	7.8 ± 2.3	7.3 ± 1.65	0.49 ± 1.26	Acceptable	Lower variability in cuff-based methods	Reshetnik et al.
*SphygmoCor* Mobil-O-Graph	102 (29.4% women)	65 ± 13	—	ITM	1^st^ measurement 10.0 ± 2.3 2^nd^ measurement 9.8 ± 2.3	1^st^ measurement 10.4 ± 2.8 2^nd^ measurement 10.6 ± 2.8	—	Not assessable	Lower variability in cuff-based methods	Grillo et al.
Mobil-O-Graph	649 (49.4% women)	47.7 ± 14.2	28.8 ± 5.8	—	7.5 ± 1.7	—	—	Not assessable	Systolic blood pressure affects the PWV results	Silva et al.
*SphygmoCor* Arteriograph	35	41	27.3	ITM	—	—	—	Not assessable	MAP affects the PWV and cfPWV results; however, oscillometric depends more on: age, height, sex and current heart rate	Podrug et al.

**Table 8:** 2010 Artery Society guidelines for validation of pulse wave velocity measurement devices.

Measurement requirements for reference standards
Either invasive (catheter-based) or noninvasive (tonometry-based) validation can be performed.
Proximal and distal measurement sites must be equal between reference standard and device under test.
Ideal: simultaneous recording using 2 catheters/tonometers; alternative: single catheter/tonometer using ECG gating.
For invasive validation: ideal: high-fidelity pressure tip catheters; alternative: fluid-filled catheters.
Recording sampling rate of at least 1 kHz.
Transit time should be determined from the waveforms using an intersecting tangent algorithm
Pulse transit time averaged over at least 10 cardiac cycles

**Subject selection criteria for validation studies**

Sample size: at least 90 subjects (but a minimum of 83 complete datasets to allow for an ∼5% dropout rate)
Sex distribution: ≥ 40% men and ≥ 40% women
Age distribution: a minimum of 25 individuals per age group (< 30, 30–60, > 60 years)

**Exclusion criteria**

Not in sinus rhythm
Pacemaker dependent
Pregnant
Body mass index ≥ 30 kg/m^2^
Known significant carotid or femoral artery stenosis
Age under 18 years (except when validating in children)

**Table 9:** 2024 Artery Society guidelines for validation of pulse wave velocity measurement device.

Measurement requirements for reference standards
Either invasive (catheter-based) or noninvasive (tonometry-based) validation can be performed.
Proximal and distal measurement sites must be equal between reference standard and device under test.
Ideal: Simultaneous recording using 2 catheters/tonometers; alternative: Single catheter/tonometer using ECG gating.
For invasive validation: ideal: high-fidelity pressure tip catheters; alternative: fluid-filled catheters.
Recording sampling rate of at least 120 Hz.
Foot detection using intersecting tangent or diastole patching algorithm.
Pulse transit time averaged over at least 10 cardiac cycles

**Subject selection criteria for validation studies**

Sample size: ≥85 complete datasets (suggested recruitment *n* of ≥ 90 to allow for 5% dropout)
Sex distribution: ≥ 40% men and ≥ 40% women
Age distribution: ≥ 10 individuals in each of the following classes: < 30, 30–49, 50–69, and ≥ 70
PWV distribution: ≥ 5% of the reference PWV readings ≤ 6 m/s, ≥ 5% with ≥ 10 m/s, and ≥ 20% with ≥ 8 m/s

**Exclusion criteria**

Not in sinus rhythm
Pacemaker dependent
Pregnant
Body mass index ≥ 40 kg/m^2^
Clinically relevant arterial stenosis between the 2 sites of measurement
Severe aortic valve stenosis
Age under 18 years (except when validating in children)
Specific criteria (if any) for the devices used

## Data Availability

Data are available upon request from the corresponding author.

## Supporting Information

This systematic review contains the quality assessment results of the studies included into analysis. Newcastle–Ottawa scale adapted for cross-sectional studies was used for evaluation of the quality. Each study was judged on seven elements, categorized into three groups: the selection of the study groups, the comparability of the groups, and the ascertainment of either the exposure or outcome of interest for case-control or cohort studies, respectively. Stars are awarded for each quality element and provide a quick visual assessment. The highest quality studies are awarded up to 10 stars.
