# Supplementary material for: Evaluation of Arterial Stiffness Parameters Measurement With Noninvasive Methods—A Systematic Review
Source: Cardiol Res Pract. 2024 Dec 19;2024:4944517. doi: 10.1155/crp/4944517 (PMC11671637; doi:10.1155/crp/4944517)
Supplement: Supporting Information — Additional supporting information can be found online in the Supporting Information section. [file 4944517.f1.docx]

|  | Selection | Comparability | Outcome | Total |
| --- | --- | --- | --- | --- |
| Salvi et al. | ** | * | *** | 7 |
| Weber et al. | ** | * | ** | 6 |
| Weber et al. | *** | * | ** | 7 |
| Walser et al. | ** | * | ** | 5 |
| Hametner et al. | ** | * | ** | 5 |
| Stabouli et al. | ** | * | ** | 5 |
| Hwang et al. | ** | ** | ** | 6 |
| Bultin et al. | **** | * | *** | 8 |
| Kolkenbeck-Ruh et al. | **** | ** | ** | 8 |
| Berukstis et al. | *** | * | ** | 6 |
| Van Dijk et al. | *** | * | ** | 6 |
| Del Giorno et al. | *** | * | *** | 7 |
| Bia et al. | ** | * | ** | 5 |
| Safardis et al. | *** | * | ** | 6 |
| Vaios et al. | ** | * | * | 4 |
| Vaios et al. | ** | * | ** | 5 |
| Souza& Weimar | * | * | * | 3 |
| Schwartz et al. | *** | ** | * | 6 |
| Staef et al. | ** | ** | ** | 6 |
| Reshetnik et al. | *** | * | ** | 6 |
| Grillo et al. | *** | * | *** | 7 |
| Silva et al. | *** | ** | ** | 7 |
| Podrug et al. | ** | * | ** | 5 |

**NEWCASTLE - OTTAWA QUALITY ASSESSMENT SCALE (adapted for cross sectional studies)**

**NEWCASTLE - OTTAWA QUALITY ASSESSMENT SCALE**

**(adapted for cross sectional studies)**

**Selection: (Maximum 5 stars)**

1) Representativeness of the sample:

1. Truly representative of the average in the target population. * (all subjects or random

sampling)

1. Somewhat representative of the average in the target population. * (non-random
2. sampling)
3. Selected group of users.
4. No description of the sampling strategy.

2) Sample size:

1. Justified and satisfactory. *
2. Not justified.

3) Non-respondents:

1. Comparability between respondents and non-respondents characteristics is established,
2. and the response rate is satisfactory. *
3. The response rate is unsatisfactory, or the comparability between respondents and nonrespondents is unsatisfactory.
4. No description of the response rate or the characteristics of the responders and the nonresponders.

4) Ascertainment of the exposure (risk factor):

1. Validated measurement tool. **
2. Non-validated measurement tool, but the tool is available or described.*
3. No description of the measurement tool.

**Comparability: (Maximum 2 stars)**

1) The subjects in different outcome groups are comparable, based on the study design or analysis. Confounding factors are controlled.

1. The study controls for the most important factors (age, BMI and obstetric history). *
2. The study control for any additional factor (e.g. race/ethnicity, socioeconomic status,
3. smoking status, drinking, hobbies, history of illness, sexual relationships) *

**Outcome: (Maximum 3 stars)**

1) Assessment of the outcome:

1. Independent blind assessment. **
2. Record linkage. **
3. Self-report. *
4. No description.

2) Statistical test:

1. The statistical test used to analyze the data is clearly described and appropriate, and the measurement of the association is presented, including confidence intervals and the probability level (p value). *
2. The statistical test is not appropriate, not described or incomplete.

**Scores for cross-sectional studies**

Very good studies: 9-10 points

Good studies: 7-8 points

Satisfactory studies: 5-6 points

Unsatisfactory studies: 0 to 4 points
